# Brain blood vessel autoantibodies in patients with NMDA and GABA_A_ receptor encephalitis: identification of unconventional Myosin-X as target antigen

**DOI:** 10.3389/fncel.2023.1077204

**Published:** 2023-01-30

**Authors:** Lucie Y. Li, Jakob Kreye, Malgorzata Burek, César Cordero-Gomez, Paula C. Barthel, Elisa Sánchez-Sendín, Hans-Christian Kornau, Dietmar Schmitz, Madeleine Scharf, Patrick Meybohm, S. Momsen Reincke, Harald Prüss, Markus Höltje

**Affiliations:** ^1^Institute of Integrative Neuroanatomy Berlin, Charité-Universitätsmedizin Berlin, corporate member of Freie Universität Berlin, Humboldt-Universität zu Berlin and Berlin Institute of Health, Berlin, Germany; ^2^German Center for Neurodegenerative Diseases (DZNE) Berlin, Berlin, Germany; ^3^Department of Neurology and Experimental Neurology, Charité-Universitätsmedizin Berlin, corporate member of Freie Universität Berlin, Humboldt-Universität Berlin and Berlin Institute of Health, Berlin, Germany; ^4^Department of Pediatric Neurology, Charité-Universitätsmedizin Berlin, corporate member of Freie Universität Berlin and Humboldt-Universität zu Berlin and Berlin Institute of Health, Berlin, Germany; ^5^Department of Anaesthesiology, Intensive Care, Emergency and Pain Medicine, University Hospital Würzburg, Würzburg, Germany; ^6^Charité-Universitätsmedizin Berlin, corporate member of Freie Universität Berlin, Humboldt- Universität zu Berlin and Berlin Institute of Health, Neuroscience Research Center, Berlin, Germany; ^7^Max Delbrück Center for Molecular Medicine in the Helmholtz Association, Berlin, Germany; ^8^Einstein Center for Neurosciences Berlin, Berlin, Germany; ^9^Bernstein Center for Computational Neuroscience Berlin, Berlin, Germany; ^10^Institute of Experimental Immunology, EUROIMMUN AG, Lübeck, Germany

**Keywords:** blood-brain barrier, autoimmunity, encephalitis, occludin, Myosin-X

## Abstract

**Introduction:** The antibody repertoire from CSF-derived antibody-secreting cells and memory B-cells in patients with encephalitis contains a considerable number of antibodies that do not target the disease-defining autoantigen such as the GABA or NMDA receptors. This study focuses on the functional relevance of autoantibodies to brain blood vessels in patients with GABA_A_ and NMDA receptor encephalitis.

**Methods:** We tested 149 human monoclonal IgG antibodies from the cerebrospinal fluid of six patients with different forms of autoimmune encephalitis on murine brain sections for reactivity to blood vessels using immunohistochemistry. Positive candidates were tested for reactivity with purified brain blood vessels, effects on transendothelial electrical resistance (TEER), and expression of tight junction proteins as well as gene regulation using human brain microvascular endothelial hCMEC/D3 cells as *in vitro* blood-brain barrier model. One blood-vessel reactive antibody was infused intrathecally by pump injection in mice to study *in vivo* binding and effects on tight junction proteins such as Occludin. Target protein identification was addressed using transfected HEK293 cells.

**Results:** Six antibodies reacted with brain blood vessels, three were from the same patient with GABA_A_R encephalitis, and the other three were from different patients with NMDAR encephalitis. One antibody from an NMDAR encephalitis patient, mAb 011-138, also reacted with cerebellar Purkinje cells. In this case, treatment of hCMEC/D3 cells resulted in decreased TEER, reduced Occludin expression, and mRNA levels. Functional relevance *in vivo* was confirmed as Occludin downregulation was observed in mAb 011-138-infused animals. Unconventional Myosin-X was identified as a novel autoimmune target for this antibody.

**Discussion:** We conclude that autoantibodies to blood vessels occur in autoimmune encephalitis patients and might contribute to a disruption of the blood-brain barrier thereby suggesting a potential pathophysiological relevance of these antibodies.

## Introduction

Autoantibodies associated with neurological diseases have deeply changed the clinical landscape and our understanding of immunological processes in the nervous system. Especially antibodies against neuronal surface receptors turned out to be directly pathogenic, hallmarking previously unclassified disease entities. In anti-N-methyl-D-aspartate receptor (NMDAR) encephalitis, antibodies targeting the NR1 subunit reduce surface NMDA receptor clusters and disrupt synaptic currents (Hughes et al., 2010; Kreye et al., 2016). Patients develop psychiatric symptoms typically involving behavioral changes, catatonia, hallucination as well as autonomic fluctuations and seizures in the course of the disease (Dalmau et al., 2007). The more recently discovered anti-γ-aminobutyric acid A receptor (GABA_A_R) encephalitis is characterized by antibodies reducing the synaptic and extra-synaptic density of GABA_A_ receptors and exerting electrophysiological changes in cultured neurons (Ohkawa et al., 2014; Petit-Pedrol et al., 2014; Pettingill et al., 2015). Patients characteristically present with catatonia, seizures, refractory status epilepticus, cognitive impairment, and MRI abnormalities (Ohkawa et al., 2014; Petit-Pedrol et al., 2014; Pettingill et al., 2015; Spatola et al., 2017).

Despite the advances in unraveling the molecular mechanisms of anti-NMDAR and anti-GABA_A_R antibodies, knowledge about how they gain access to central nervous system (CNS) targets is still lacking. Possible mechanisms of immune system components transmigrating the blood-brain barrier (BBB) have been investigated in other inflammatory autoimmune disorders. In neuropsychiatric systemic lupus erythematodes (NPSLE), BBB disruption has been demonstrated to be a crucial step in disease development (Kowal et al., 2004; Huerta et al., 2006; Hirohata et al., 2014). This process is fueled by endothelial antibody binding and upregulating the expression of proinflammatory cytokines and leukocyte adhesion molecules (Meroni et al., 2003; Armitage et al., 2004; Yoshio et al., 2013). Furthermore, for neuromyelitis optica (NMO) it has been shown that BBB disruption correlates with disease severity (Tomizawa et al., 2012). Findings in NMO have uncovered monoclonal antibodies targeting Glucose-regulated protein 78 (GRP78), which after repeated administration, caused extravasation of serum albumin, IgG, and fibrinogen in mouse brains (Shimizu et al., 2017). Shortly after, GRP78 antibodies were also discovered to impair the BBB in patients with paraneoplastic cerebellar degeneration with Lambert-Eaton myasthenic syndrome (PCD-LEMS), thereby potentially allowing access of pathogenic autoantibodies (Shimizu et al., 2019). Thus, antibodies targeting blood vessels can be directly pathogenic by inducing an endothelial pro-inflammatory phenotype, can cause BBB dysfunction, and possibly even promote the transition of macromolecules through the BBB.

Additionally, studies of recombinant human monoclonal antibodies (mAbs) have demonstrated that recombinant mAbs from CSF-derived antibody-secreting cells and memory B-cells especially in patients with NMDAR encephalitis do not only target the disease-defining autoantigen (Kreye et al., 2016, 2021). Rather, the majority of antibodies strongly react with further brain epitopes. Their possible involvement in the disease pathomechanism remains unclear. Hence, with our non-biased approach using recombinant production of CSF-derived mAbs (Kreye et al., 2016, 2021), we aimed to further investigate the intrathecal human monoclonal antibody repertoire. Using immunofluorescence methods on murine brain tissue we identified a subgroup of blood vessel reactive mAbs to a similar extent in patients diagnosed with NMDAR encephalitis and GABA_A_R encephalitis.

To our knowledge, this is the first characterization of autoantibodies to brain blood vessels in NMDAR encephalitis and GABA_A_R encephalitis patients. We illustrate binding to brain blood vessels *in vitro* and replicate characteristic binding *in vivo*. Furthermore, in this qualitative study, we identified Myosin-X as the target antigen for one selected mAb and showed its functional effects *in vitro* and *in vivo*. Collectively, we provide the first evidence for a putative contribution of brain blood vessel reactive mAbs to disease development.

## Materials and methods

### Immunohistochemistry on mouse brain sections

For tissue sections of unfixed mice brains, animals were sacrificed, brains were removed and snap-frozen in −50°C cold 2-methyl butane. Twenty micrometer sagittal sections were cut and processed as described previously (Kreye et al., 2016, 2021).

### Indirect immunofluorescence assay on monkey and rat brain sections

Stainings were performed using slides with a biochip screening array of brain tissue cryosections (cerebellum of rat and *Macaca mulatta*). Each biochip mosaic was incubated with 35 μl of PBS-diluted sample at 4°C for 16 h in a humidity chamber. In the second step, Alexa488-labelled goat anti-human IgG (Jackson Research, Suffolk, United Kingdom), was applied and incubated at RT for 2 h. Results were evaluated independently by two observers using a fluorescence microscope (EUROStar II, Euroimmun AG, Lübeck, Germany).

### Generation of human monoclonal antibodies

Human monoclonal antibodies were previously generated as recombinant proteins from patients with NMDA receptor encephalitis or GABA_A_ receptor encephalitis (Kreye et al., 2016, 2021; Nikolaus et al., 2018). The diagnosis was confirmed by: (i) the presence of autoantibodies against the respective autoantigen in the patient’s cerebrospinal fluid as detected in a commercial cell-based assay (EUROIMMUN, Lübeck Germany); and by (ii) typical neurological symptoms. For monoclonal antibody isolation, we used established methods (Kreye et al., 2016, 2020; 2021; Reincke et al., 2020). In brief, from patients’ cerebrospinal fluid, single antibody-secreting cells and B cells were isolated using fluorescence-activated cell sorting. From single-cell cDNA variable immunoglobulin encoding genes were amplified, sequenced, and cloned into expression vectors. Pairs of functional heavy and light chain vectors were used to transfect human embryonic kidney (HEK293T) cells using Polyethylenimine (Polysciences, Inc., Warrington, USA). On day three/four and day seven after transfection cell culture supernatants were harvested, then centrifuged at 2,000× *g* for 5 min at 4°C to remove cell debris before sodium acid was added to a concentration of 0.05% to prevent bacterial growth. The human IgG concentration in cell culture supernatants was determined using a commercial ELISA kit (Mabtech, Nacka Strand, Sweden) following the provider’s instructions. For functional assays, mAbs were purified from supernatants using Protein G Sepharose beads (GE Healthcare), then dialyzed against PBS and sterile-filtered using 0.2 μm filter units (GE Healthcare). Recombinant mGO53 antibody served as a control antibody in *in vitro* and *in vivo* experiments.

### Commercial antibodies

A monoclonal anti-Myosin-X antibody was purchased from Santa Cruz Biotechnology (# sc-166720, St. Cruz, USA). A monoclonal anti-smooth muscle actin (SMA) antibody was from Agilent Dako (#M0851, Santa Clara, USA). A rat monoclonal anti-CD31 antibody was from BD Biosciences (#553708, Franklin Lakes, NJ, USA). A monoclonal anti-CD34 antibody was from Arigo Biolaboratories (#ARG52756, Hsinchu City, Taiwan). A polyclonal anti-Collagen IV antibody was purchased from Abcam (#ab6586, Cambridge, UK). Polyclonal anti-Occludin and anti-Claudin5 antibodies were from Thermo Fisher Scientific (#71-1500 and #34-1600; Waltham, USA). A monoclonal anti-VE-cadherin antibody was from Cell Signaling (#2500, Danvers, USA). A mouse monoclonal anti-ZO-1 antibody was from Thermo Fisher Scientific (#33-9100).

### Cell culture of human cerebral endothelial cells

Immortalized human cerebral microvascular endothelial cells hCMEC/D3 (Weksler et al., 2005) obtained from CELLutions Biosystems Inc. (#CLU512, Burlington, Ontario, Canada) were grown to confluence on coverslips. Cells were then used for either immunofluorescence staining on fixed cells or live incubation. For the fixed approach, endothelial cells were incubated with 4% PFA for 10 min and subsequently washed twice with PBS. Primary human antibodies (5 μg/ml) remained on the coverslips overnight at 4°C. For live incubation, a patient antibody was added to the medium at 5 μg/ml overnight at 36°C and 5% CO_2_. After fixation with 4% PFA for 20 min cells were incubated with a secondary antibody for 2 h at RT in the dark.

### Transfection

Human embryonic kidney (HEK) 293 cells were cultured in 24-well multiplates to 70% confluency and transfected with 1 μg plasmid cDNA coding for eGFPC1-hMyoX (Addgene, #47608) per well for 24 h using Polyethylenimine as transfection reagent. Transfected cells were fixed with 4% paraformaldehyde for 20 min at 4°C. Cells were subsequently permeabilized with 0.1% Triton X-100. Thereafter, cells were incubated with commercial antibodies at indicated concentrations or human monoclonal antibodies at 5 μg/ml.

### Purification of mouse brain vessels

Purification of mouse brain vessels was performed following a previously described protocol (Boulay et al., 2015). In brief, myelin was removed from adult mice brain homogenate using an 18% dextran solution and density gradient centrifugation. From the resulting suspension brain blood vessels between 30 and 100 μm and >100 μm were collected by a sequence of filtering steps and directly used for immunohistochemistry or lysed for further analysis.

### Measurement of TEER (transendothelial electrical resistance)

Human hCMEC/D3 cells were seeded onto Matrigel-coated trans-well inserts (0.4 μm pore size, Corning) at a density of 40 × 10^3^ in ECM Medium (PELOBiotech, Martinsried, Germany) supplemented with 5% FCS. After 5 days ECM was depleted of growth factors and FCS was reduced to 0.5% for differentiation. Subsequently, endothelial cells were treated with patient antibodies at the indicated concentrations or left untreated for an additional 24 or 48 h. TEER measurements across each trans-well were conducted using chopstick electrodes (STX-PLUS, WPI, Sarasota, Fl, USA) and an Epithelial-Volt/Ohm-Meter (EVOM3, WPI). The TEER values of blank filters were subtracted from the measured values before calculations. Values were measured in triplicates.

### Real-time PCR

RNA was isolated using the NucleoSpin^®^ RNA Isolation Kit (Machery-Nagel, Düren, Germany) according to the manufacturer’s instructions. Total RNA (1 μg) was reverse transcribed using the High Capacity cDNA Reverse Transcription Kit (Thermo Fisher Scientific). The following TaqMan probes (Thermo Fisher Scientific) were used: Hs00901465_m1 (CDH5, VE-cadherin), Hs01558409_m1 (CANX, Calnexin), Hs00533949_s1 (CLDN5, Claudin5), Hs00202485_m1 (MYO10, Myosin-X), Hs00170162_m1 (OCLN, Occludin), Hs01551861_m1 (TJP1, ZO-1) with the TaqMan^®^ Fast Advanced Master Mix in the QuantStudio^TM^ 7 Flex Real-Time PCR System (Thermo Fisher Scientific). CANX was used as an endogenous control. The relative expression was calculated using the comparative Ct method with QuantStudio^TM^ Real-Time PCR Software v1.7.1.

### Membrane preparations

hCMEC/D3 cells were grown to confluency, seeded in 6-well plates, and cultured for 48 h with the respective antibodies as indicated. Following the removal of medium, the cells were washed with PBS, harvested, homogenized, and lysed in a glass/teflon homogenizer under hypoosmotic conditions. Homogenates were centrifuged at 1,043× *g* for 10 min to obtain a postnuclear supernatant. The resulting supernatant was centrifuged at 267,008× *g* for 30 min to obtain highly enriched cellular membranes. Membrane fractions were subjected to immunoblot analysis.

### Immunoprecipitation

Triton-X100 (1%) lysate from monkey (*Macaca mulatta)* cerebella was centrifuged at 21,000× *g* at 4°C for 15 min and clear supernatants were incubated with the patient‘s serum (diluted 1:33) at 4°C for 3 h. The samples were then incubated with Protein G Dynabeads (ThermoFisher Scientific, Dreieich, Germany) at 4°C overnight to capture immunocomplexes. The beads were washed 3 times with PBS and eluted with NuPage LDS sample buffer (ThermoFisher Scientific) containing 25 mmol/L dithiothreitol at 70°C for 10 min. Carbamidomethylation with 59 mM iodoacetamide (Bio-Rad, Hamburg, Germany) was performed prior to SDS-PAGE (NuPAGE, ThermoFisher Scientific). Separated proteins were visualized with Coomassie Brillant Blue (G-250; Merck), and identified by mass spectrometric analysis or were applied for Western Blot.

### Intrathecal antibody infusion

Experimental animals were randomized for the different treatment groups by an independent investigator. 10–12 weeks old C57BL6/J mice received either mAb 011-138 or control mAb mGO53 (100 μg over 7 days, 200 μg over 14 days, 1 μg/h). Antibody cerebroventricular infusion was performed using osmotic pumps (model 1002, Alzet, Cupertino, CA). Pump characteristics included: volume 100 μl and flow rate 0.25 μl/h. For pump implantation, mice were placed in a stereotaxic frame and a cannula was inserted into the right ventricle (coordinates: 0.2 mm posterior and ± 1.00 mm lateral from bregma, depth 2.2 mm). The cannula was connected to a pump, which was subcutaneously implanted in the interscapular space of the animals. After pump implantation, the animals were daily monitored to assess clinical symptoms and weight variations. Mice were sacrificed either on day 7 or day 14, brains were removed and snap-frozen in 2-methylbutan for immunohistochemistry. In addition, blood samples were collected for serum preparation (2,000× *g* for 15 min, RT). Unfixed sections from mouse brains were either incubated with FITC-coupled anti-human IgG secondary antibody alone or serum from treated mice prior to the application of secondary antibody.

### Fluorescence intensity measurements

To analyze the intensity of IgG binding to the brain blood vessel images were taken at 40× magnification using a Leica DMLB epifluorescence microscope. Image areas attributed to vessel walls were cropped and average gray scale brightness values were calculated by the histogram function of Adobe Photoshop CS6 software.

## Results

### Autoimmunity to brain blood vessels and Purkinje cells

We have previously reported that the CSF antibody repertoire from patients with NMDAR encephalitis and GABA_A_R encephalitis does not only include mAbs that are autoreactive to the disease-defining antigen. Besides neuronal and glial binding, several CSF antibodies exhibit autoreactivity to brain blood vessels with yet unknown functional relevance. Here, we systematically screened 149 CSF-derived mAbs from six autoimmune encephalitis patients, including 67 from GABA_A_R encephalitis, 61 from NMDAR encephalitis, and 21 from non-GABA_A_R/non-NMDAR encephalitis patients for blood vessel autoreactivity in murine brain tissue. We identified six mAbs with prominent brain blood vessel reactivity, of which three (113-109, 113-111, 113-126) had been isolated from a GABA_A_R encephalitis patient (Nikolaus et al., 2018; Kreye et al., 2021) and three (080-221, 003-151, 011-138) from NMDAR encephalitis patients (Kreye et al., 2016). Patient details can be obtained from these previous publications.

**Figure 1:**
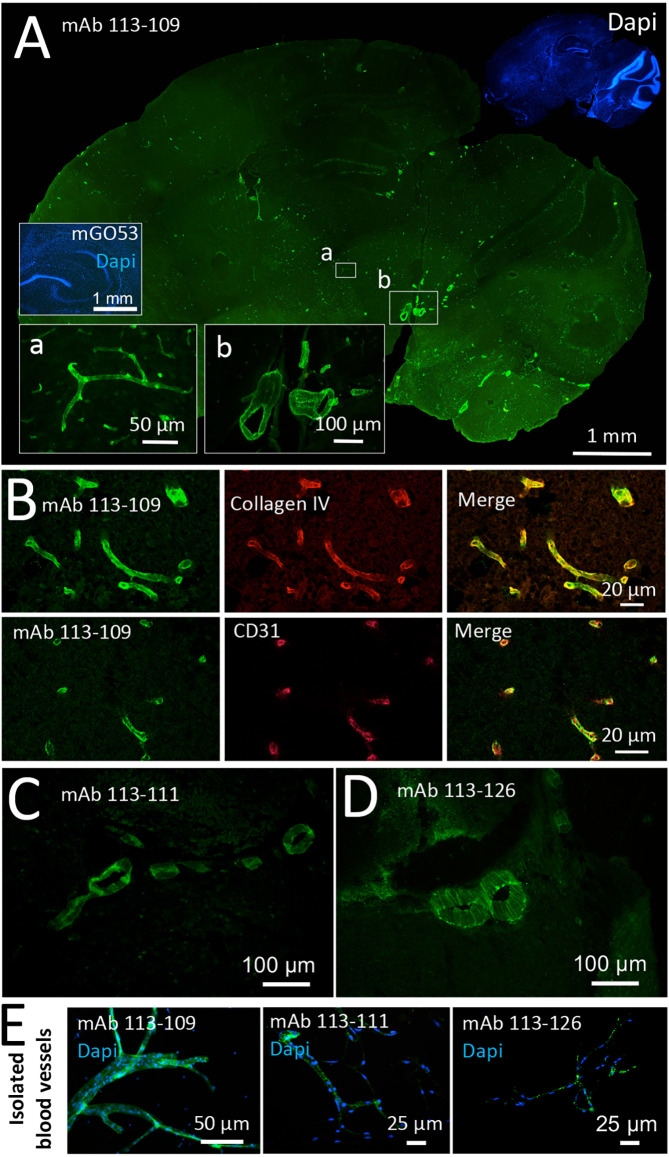
Recombinant monoclonal antibodies from a GABA_A_R encephalitis patient bind to brain blood vessels. Sections from unfixed and unpermeabilized adult mouse brains were incubated with 5 μg/ml of the respective monoclonal antibodies obtained from a patient diagnosed with GABA_A_R encephalitis. Visualization of tissue binding was performed using a FITC-coupled anti-human IgG secondary antibody. **(A)** Sagittal brain section incubated with human monoclonal antibody (mAb) 113-109. Prominent staining of blood vessels of all diameters in all brain regions was obtained (see insets a and b). Incubation with control mAb mGO53 yielded no tissue staining (inset). **(B)** Double stainings against Collagen IV (upper panel) and CD31 (lower panel) confirm immunoreactivity of mAb 113-109 to blood vessels. **(C)** Sagittal brain section incubated with mAb 113-111. Prominent staining of large blood vessels was obtained in all brain regions (the area between the cerebellum and midbrain is shown). **(D)** Sagittal brain section incubated with mAb 113-126. Again, prominent staining of large blood vessels in all brain regions was obtained (the area between the cerebellum and midbrain is shown). **(E)** Stainings of isolated brain blood vessels. Blood vessels were obtained from homogenized mouse brains by combinatory centrifugation and filtering steps. A 30-100 μm filter size fraction incubation with all three antibodies resulted in clear staining of the vessels within this fraction. Note that the diameter of mounted blood vessels is subject to shrinking artifacts during the staining procedure.

Among the three blood vessel reactive antibodies obtained from one young patient with GABA_A_R encephalitis, mAb 113–109 provided remarkably strong staining of vessels of all diameters throughout the entire brain (Figure 1A). The homogeneous staining of mAb 113–109 throughout brain blood vessels of all sizes suggested an epitope structure present up to the capillary level. Blood vessel staining was further confirmed using co-stainings against the basal membrane constituent Collagen IV and the blood vessel endothelial cell marker CD31 and is shown exemplarily (Figure 1B). In contrast, mAbs 113-111 and 113-126 obtained from the same patient stained primarily large-sized vessels (Figures 1C,D, see also Supplementary Figure 1 for co-stainings with CD31). Additionally, in blood vessels isolated from adult mouse brains, all three antibodies showed clear staining on the 30–100 μm fraction of brain blood vessels (Figure 1E).

**Figure 2:**
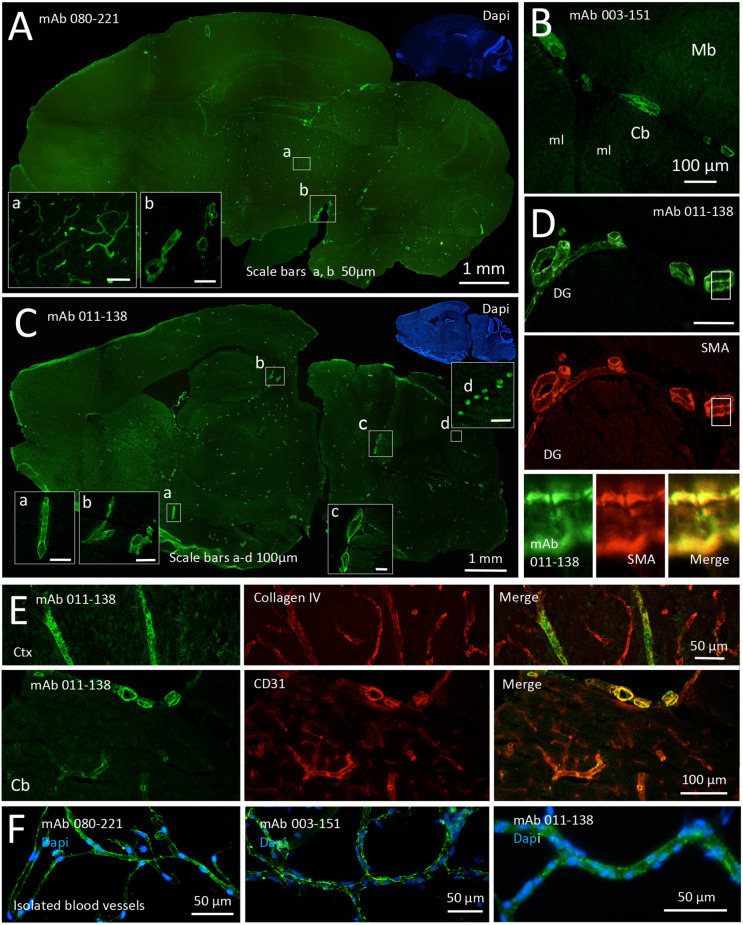
Recombinant monoclonal antibodies from NMDAR encephalitis patients bind to brain blood vessels and Purkinje cells. Unfixed and unpermeabilized sections from adult mouse brains were incubated with 5 μg/ml of the respective human monoclonal antibodies (mAbs) obtained from three different patients diagnosed with NMDAR encephalitis. Visualization of tissue binding was performed using a FITC-coupled anti-human IgG secondary antibody. **(A)** Sagittal brain section incubated with mAb 080-221. Prominent staining of large to small blood vessels including capillaries was obtained in all brain regions (see insets a and b). **(B)** Sagittal brain section incubated with mAb 003-151. Prominent staining of large to mid-size blood vessels in all brain regions was obtained (a section of cerebellum, *Cb*, and midbrain, *Mb*, is shown; *ml* molecular layer). **(C)** Sagittal brain section incubated with mAb 011-138. Prominent staining of large to mid-size blood vessels in all brain regions was obtained (see insets a, b, and c). In addition to blood vessel staining cerebellar Purkinje cells showed marked somatic staining (see inset d). **(D)** Double staining against human IgG and SMA (smooth muscle actin) in a brain section adjacent to the dentate gyrus (*DG*) of the hippocampus formation. Bound human IgG was mainly detected within the SMA-positive muscle layer of the vessels. **(E)** Double stainings against Collagen IV (upper panel) and CD31 (lower panel) confirm immunoreactivity of mAb 011-138 to blood vessels*. Ctx Cortex* **(F)** Staining of isolated brain blood vessels. Blood vessels were obtained from homogenized mouse brains by combinatory centrifugation and filtering steps. A 30–100 μm filter size fraction incubation with all three antibodies resulted in clear staining of the vessels within this fraction. Note that the diameter of mounted blood vessels is subject to shrinking artifacts during the staining procedure.

An additional three blood vessel reactive mAbs were detected among the recombinant mAbs derived from the CSF of anti-NMDAR encephalitis patients. These include mAb 080-221 which showed prominent staining of vessels of all sizes including the capillaries (Figure 2A; for capillary staining, see inset a, see also Supplementary Figure 1). In contrast, the other two mAbs predominantly stained mid to large-size blood vessels. Vessels stained by mAb 003–151 showed a less homogeneous and more speckled pattern (Figure 2B and Supplementary Figure 1). In addition to the strong reactivity to brain blood vessels (Figure 2C), mAb 011-138 reacted with cerebellar Purkinje cells, leading to pronounced somatic staining (Figure 2C, inset d). To further characterize mAb 011-138 reactivity, we double-stained with smooth muscle actin (SMA) on murine brain slices (Figure 2D). The mAb 011-138 signal exhibited a high degree of overlap with SMA-positive smooth muscle cells of the vessels supporting our previous observation that the patient mAb predominantly recognizes large to mid-size vessels. Again, Collagen IV and CD31 stainings were applied to mark blood vessels (Figure 2E). Additionally, reactivity to brain blood vessels was confirmed using purified murine brain vessels sized 30–100 μm (Figure 2F).

In this cohort of mAbs, antibody 011-138 stood out due to its combinatory reactivity to brain blood vessels and a defined group of neuronal cells—cerebellar Purkinje cells, making mAb 011-138 a particularly interesting candidate for further investigation in terms of target identification as well as possible mechanistic effects.

### Monoclonal antibody 011-138 reduces TEER in an *in vitro* BBB model and decreases the expression of cell junction protein occludin

**Figure 3:**
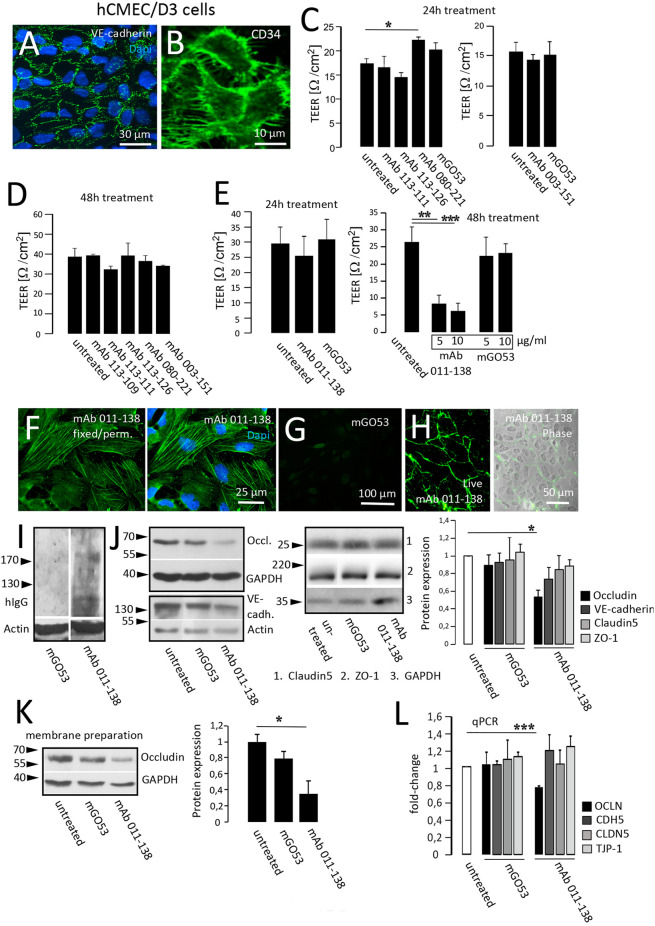
Antibody 011-138 decreases transendothelial electrical resistance and downregulates tight junctional Occludin. **(A,B)** Human cerebral microvascular endothelial cells (hCMEC/D3) were used as an *in vitro* blood-brain barrier model. Tight junction protein VE-cadherin and the endothelial cell marker CD34 were clearly expressed and depicted adjacent cell boundaries. The following functional experiments were carried out on live cells grown to confluence. **(C)** After 24 h of antibody treatment at 5 μg/ml, none of the human monoclonal antibodies (mAb) decreased TEER, only mAb 080-221 increased TEER. **(D)** No effects on TEER were observed after incubation for 48 h with 5 μg/ml with the respective antibodies. **(E)** Incubation with 5 μg/ml mAb 011-138 for 24 h had no significant effects on TEER. Conversely, incubation for 48 h resulted in a highly significant reduction of electrical resistance by mAb 011-138, but not mGO53, used as control Ab. Values analyzed in triplicates are expressed as means ± SEM from a representative experiment that was at least repeated once per condition **(C–D)** or from three independent experiments **(E)**. **(F–H)** Immunofluorescence detection of mAb 011-138 binding (5 μg/ml) to either live or fixed and permeabilized hCMEC/D3 cells. Fixed cells revealed a filamentous signal of mAb 011-138, and live cells showed a rather surface localized signal. Incubation with mGO53 resulted in no signal under either condition (**G**, shown for fixed cells). **(I)** Western blotting of hCMEC/D3 cell homogenates with either mAb 011-138 or control mGo53. Bound monoclonal 011-138 expressed higher molecular weight bands with a prominent signal at or above 200 kDa, mGO53 revealed no immune signal. **(J)** Confluent hCMEC/D3 cells were incubated for 48 hwith 5 μg/ml mAb 011-138 or mGO53. Western blotting for detection of Occludin, VE-cadherin, Claudin5, and ZO-1 expression. GAPDH or Actin were used as loading control. Incubation with mAb 011-138 resulted in a significant downregulation of Occludin expression exclusively. Values adjusted to loading are expressed as means ± SEM from 4–5 independent experiments. **(K)** Membrane preparations were performed from hCMEC/D3 cells following incubation with 5 μg/ml mAb 011-138 or mGO53. Western blot analysis revealed a significant removal of Occludin from the membrane compartment largely consisting of plasma membrane fractions following incubation with mAb 011-138. Western blot values adjusted to loading are expressed as normalized means ± SEM from three independent experiments. **(L)** Additionally, quantitative RT-PCR was performed to check for alterations in gene regulation of tight junction proteins. Calnexin (CANX) was used as endogenous control. Significantly decreased mRNA levels were found for Occludin (OCLN), the other genes (CDH5 VE-cadherin; CLDN5 Claudin5, and TJP-1 ZO-1) remained unchanged. Quantitative PCR values are expressed as means ± SEM from three independent experiments. *p ≤ 0.05, **p ≤ 0.01, ***p ≤ 0.001.

To test for putative pathophysiological effects on blood vessels we applied an *in vitro* model for the analysis of BBB disruption. Human cerebral microvascular endothelial cells (hCMEC/D3; Weksler et al., 2005) represent an established model to mimic the *in vivo* phenotype of the BBB and are commonly used to investigate pathomechanisms and transport processes (Helms et al., 2016). Functional evaluation of endothelial monolayer integrity in response to antibody treatment was quantified with transendothelial electrical resistance (TEER) measurements. For our purposes, hCMEC/D3 cells were grown to confluency as exemplarily shown by fluorescent staining of adherens junction protein VE-Cadherin and endothelial cell marker CD34 (Figures 3A,B). We assessed TEER changes in response to 24 h and 48 h of treatment with patient mAbs in comparison to non-reactive control mAb mGO53 (Figures 3C–E). When hCMEC/D3 cells were exposed to mAbs obtained from the patient with GABA_A_R encephalitis as well as mAbs 080-221 and 003-151 from patients with NMDAR encephalitis, TEER values did not significantly decrease, and barrier breakdown could not be observed. For 24 h treatment with mAb 080-221 an increase in TEER was seen (Figure 3C). Conversely, the results showed that treatment with mAb 011-138 led to a significant reduction of TEER after 48 h but not after 24 h (Figure 3E). A significant reduction of TEER after 48 h was achieved using 5 μg/ml and was slightly stronger with a concentration of 10 μg/ml.

In the following, we investigated the fluorescent binding pattern of mAb 011-138 in hCMEC/D3 cells. Incubation of fixed and permeabilized cells revealed a stress fiber-like filamentous staining pattern that included the plasma membrane region not observed with control mGO53 (Figures 3F,G). Incubation of live cells with 5 μg/ml mAb 011-138 resulted in a plasma membrane-like staining of the cell periphery (Figure 3H). The reactiveness of antibody 011-138 to hCMEC/D3 cells was also tested using Western Blot analysis. Blotted cell lysates were incubated with 5 μg/ml of either mAb 011-138 or mGO53 for control (Figure 3I). Incubation with antibody 011-138 yielded a major immunoreactive band slightly above 200 kDa and a few lower bands around the 130 kDa marker. Incubation with control mAb mGO53 yielded no bands.

To investigate the underlying mechanisms of BBB disruption, the expression of barrier-constituting junctional proteins was evaluated after treatment with mAb 011-138 for 48 h in comparison to the control antibody (Figure 3J). Resulting Western blots yielded a significant decrease of Occludin expression in hCMEC/D3 cells. The decreased expression of Occludin was confirmed on the mRNA level using qPCR (Figure 3L). Downregulation of Occludin by mAb 011-138 treatment was not only observed in whole cell homogenates but was also evident for plasma membrane-enriched cell fractions (Figure 3K). VE-Cadherin, Claudin5, and Zonula occludens protein-1 (ZO-1) expression was not significantly altered. Concordantly, qPCR experiments did not show significant genetic changes (Figure 3L).

As an integral component of tight junctions, Occludin may therefore contribute to the decreased TEER.

### *In vivo* reactivity of mAb 011-138 to brain blood vessels and decreased expression of occludin

**Figure 4:**
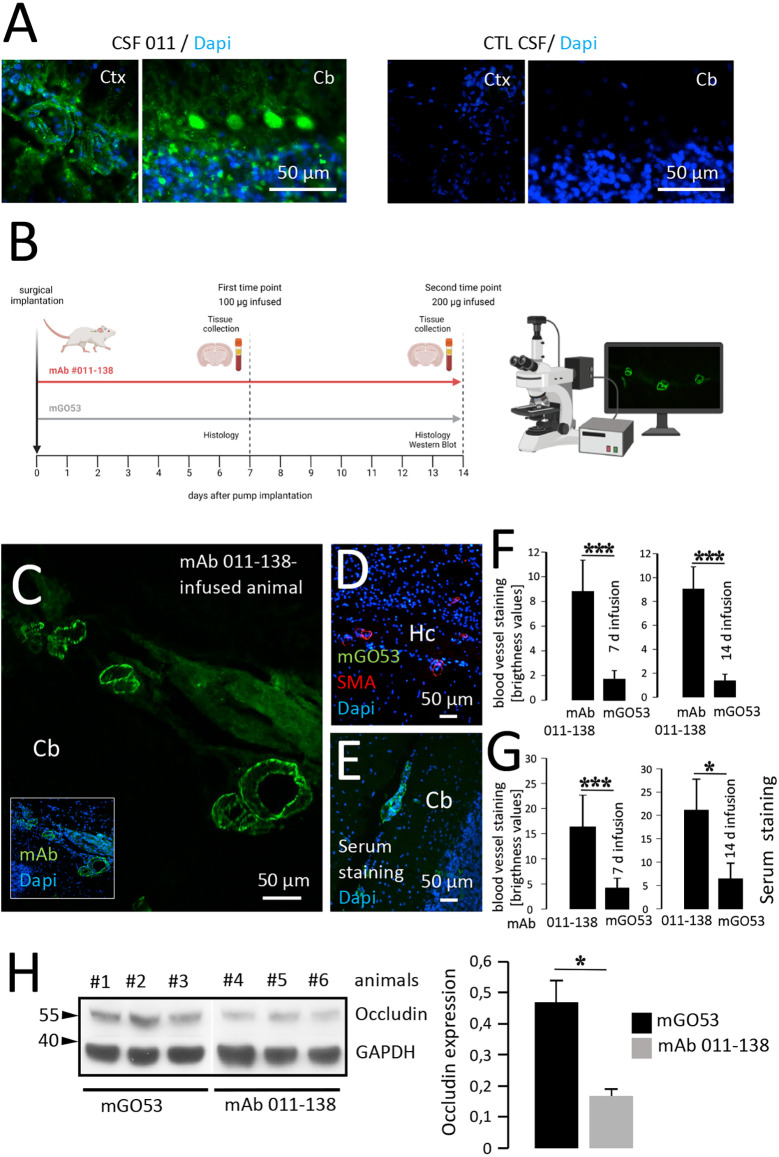
Intrathecal application of antibody 011-138 leads to *in vivo* blood vessel binding and Occludin downregulation. **(A)** Sections from unfixed adult mouse brains were incubated with CSF of patient 011 and an age-matched control patient at a dilution of 1:2. Incubation with 011 CSF resulted in IgG staining of blood vessels and cerebellar Purkinje cells. Incubation with control CSF yielded no staining. **(B)** Adult mice were either administered a dose of 100 μg of human monoclonal antibody (mAb) 011-138 into the right lateral ventricle for 7 days or 200 μg for 14 days. Animals were sacrificed, brains were removed, and immediately frozen for immunohistochemistry. In addition, blood was collected to obtain serum. **(C)** Representative sagittal brain section from an animal treated for 14 days with mAb 011-138 was incubated with FITC-coupled anti-human IgG. Clear staining of large to mid-size blood vessels in all brain regions was visible (shown for the cerebellum, *Cb*). **(D)** Sagittal brain section from one animal treated with control antibody mGO53 for 14 days. Incubation with a secondary antibody revealed no staining *Hc = hippocampus*. **(E)** Sagittal unfixed brain section from an untreated adult mouse was incubated with serum (dilution 1:200) from an animal that had received mAb 011-138 for 14 days. Visualization with secondary antibody revealed staining of larger to mid-size blood vessels by the mouse serum. **(F)** Quantification of blood vessel IgG immunoreactivity. Data are given as means ± SEM from three animals per condition. Per condition, between 38 and 43 blood vessel sections were analyzed. ***p ≤ 0.001. **(G)** Quantification of serum blood vessel IgG immunoreactivity on naive brain sections. Data are given as means ± SEM from three animals per condition. Per condition, between 22 and 45 blood vessel sections were analyzed. *p ≤ 0.05, ***p ≤ 0.001. Brightness levels of mGO53 staining were within the background range. **(H)** Downregulation of Occludin by mAb 011-138. In brains of mice treated with mAb 011-138 for 14 days protein expression was strongly reduced by 65% compared to animals that had received mGO53. Data are given as means ± SEM adjusted to loading from seven animals per condition from two independent experiments. *p ≤ 0.05. Three animals per condition from one experiment are exemplarily shown.

To first confirm the detectable presence of recombinant mAb 011-138 in patients CSF, we stained CSF-011 on unfixed murine brain sections. This showed simultaneous reactivity in vessels and Purkinje cells as well in addition to the typical NMDAR distribution (Figure 4A). Prompted by these findings and to investigate the effects of mAb 011-138 *in vivo*, mice were intrathecally infused with mAb 011-138 or isotype control using an osmotic pump system for continuous delivery into the CNS (Figure 4B). Detection of human IgG after 7 and 14 days of infusion (100 μg for 7 days and 200 μg for 14 days were administered) showed mid to large-size blood vessels staining throughout different brain regions in mAb 011-138 infused animals (exemplary vessel staining of 14-day infusion: Figure 4C). In contrast, sections of animals that received the control antibody mGO53 for infusion did not show any staining (Figures 4D,F), whilst the presence of vessels containing the target vessel wall structures was ensured by SMA-staining. From the same animals, serum was collected to check for the access of antibodies to the bloodstream to provide a source of blood vessel reactive antibodies. Staining of unfixed wild-type murine brain slices with sera from mAb 011-138 treated animals showed the same staining pattern of large to mid-size blood vessels as the previously described secondary antibody treated brain sections from animals that received mAb 011-138 intrathecally (Figures 4E,G). These findings demonstrate the capacity of antibody 011-138 to bind to its target structure *in vivo* and suggest access to the blood system from the CSF compartment in our experimental paradigm. This could represent an antibody effect as well as a lesion-induced phenomenon or be due to a physiologically occurring FcRn-mediated process.

Additionally, we conducted Western blot experiments to assess the expression of Occludin in brain tissue of animals intrathecally infused with mAb 011-138 compared to the control antibody. We found Occludin expression to be significantly decreased, matching our *in vitro* findings using hCMEC/D3 cells (Figure 4H). As already observed *in vitro*, other tight junction proteins such as VE-Cadherin, Claudin5, and ZO-1 were not significantly altered by the injection of mAb 011-138 (Supplementary Figure 2), thereby confirming the specificity of the effects on Occludin.

### Unconventional Myosin-X represents a target epitope of mAb 011-138

**Figure 5:**
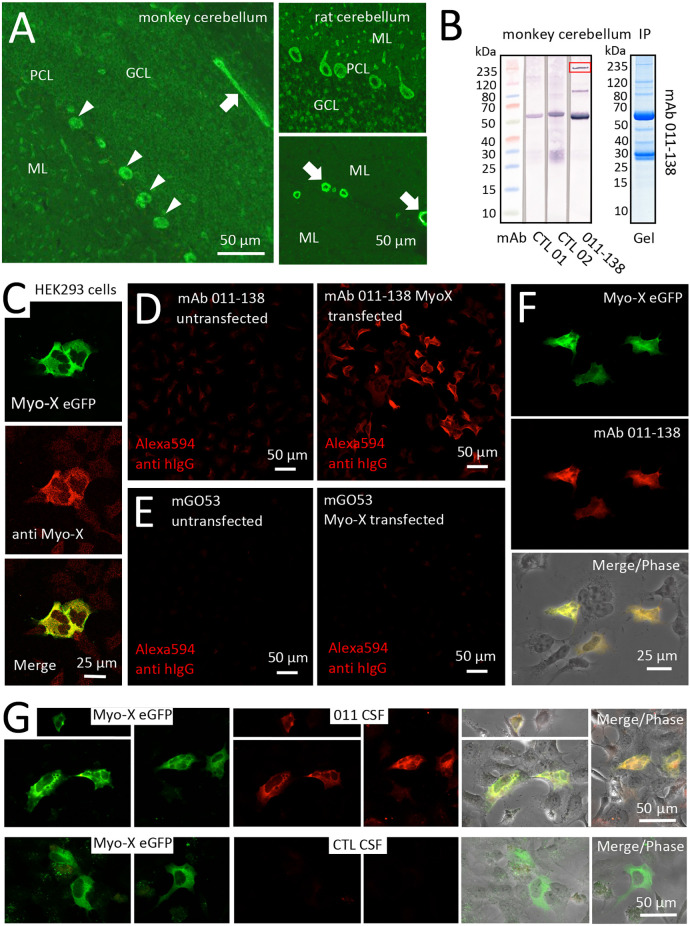
Antibody 011-138 targets Myosin-X in transfected HEK cells. **(A)** Monkey and rat brain biochip cryosections (EUROIMMUN AG) were incubated with human monoclonal antibody (mAb) 011-138 (1:100) and exhibited pronounced staining of blood vessels (left panel and lower right panel arrows) and Purkinje cells (left panel arrowheads and upper right panel). **(B)** Immunoprecipitation analysis using monkey brain lysates. Lysates were incubated with either mAb 011-138 or two other non-vessel reactive human monoclonal antibodies for control. Dynabeads were used to precipitate antibodies together with bound antigens. Elute fractions were again incubated with the precipitating antibody followed by Western blotting. The marked band at 55 kDa in all three lanes presumably resulted from detection of the precipitating heavy chain by the detecting secondary anti-human IgG conjugate. In addition, mAb 011-138 showed two distinct bands at 80 and 240 kDa (boxed), respectively. The corresponding Coomassie gel is shown for mAb 011-138. **(C)** HEK 293 cells were transfected with an eGFP construct of human Myosin-X (Myo-X) plasmid DNA for 24 h. Expression of Myo-X was verified in fixed cells using a commercial monoclonal anti-Myo-X antibody showing a very high degree of signal overlap (confocal imaging). **(D)** Incubation of Myo-X-transfected cells with 5 μg/ml of 011-138 antibody showed binding to fixed transfected cells that were absent in untransfected cells. A secondary anti-human IgG antibody coupled to Alexa594 was used for detection. **(E)** For negative control untransfected and Myo-X-transfected cells were incubated with 5 μg/ml of mGo53 antibody. No staining was observed under either condition. **(F)** In transfected cells incubated with 011-138 antibody the human IgG signal showed a very high degree of overlap with eGFP-Myo-X signal. **(G)** HEK 293 cells were transfected with an eGFP construct of human Myo-X plasmid DNA for 24 h. Incubation of Myo-X-transfected cells with patient-CSF 011 showed binding that was absent after incubation with control-CSF. The right panel shows a high degree of overlap of patient-CSF 011 signal with eGFP-Myo-X signal.

In brain biochip tissues of monkey and rat cerebellum, mAb 011-138 yielded a very similar staining pattern to the one observed in mouse brain (Figure 5A). To identify the target of mAb 011-138 we performed immunoprecipitation studies with rodent aorta lysates, purified mouse brain vessels, and monkey brain lysates as antigen-providing tissues and repeatedly received various conventional and unconventional myosins. Exemplarily, a Western blot is shown for an immunoprecipitation experiment using monkey brain lysate (Figure 5B). Incubation of the precipitated protein fraction with mAb 011-138 showed a distinct band around 240 kDa corresponding to the molecular weight of many myosin isoforms, together with a lower band around 80 kDa. Reactivity to the precipitating heavy chain around 55 kDa was shared also by other precipitating human monoclonal antibodies used for control. We were aware of the fact that myosins tend to be “sticky” and, therefore, are often pulled down unspecifically during immunoprecipitation. However, the combined occurrence of strong immunoreactivity in brain blood vessels together with the reactivity to cerebellar Purkinje cells matched the published brain distribution of unconventional Myosin-X (Sousa et al., 2006) and therefore prompted us to test for reactivity of mAb 011-138 to Myosin-X.

**Figure 6:**
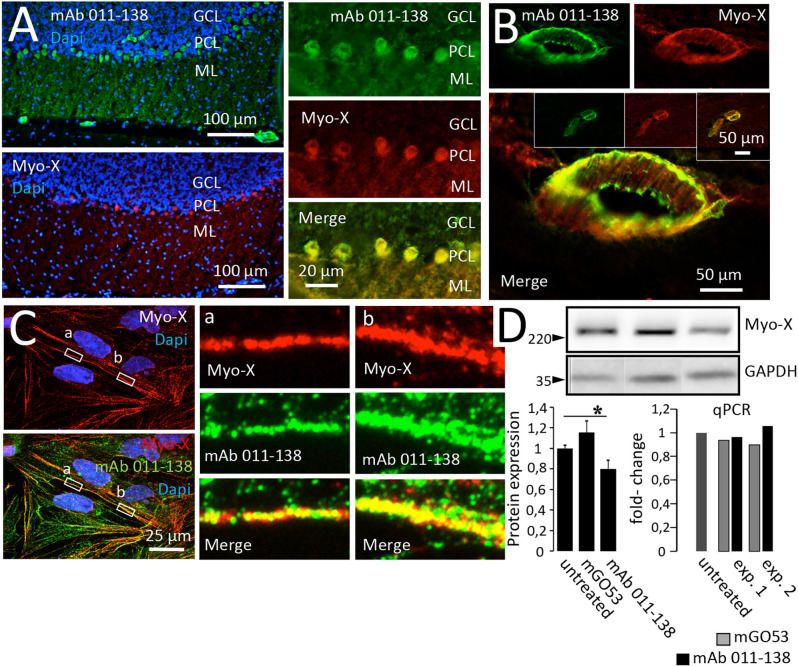
Antibody 011-138 colocalizes with commercial anti-Myosin-X antibodies in brain sections and hCMEC/D3 cells and downregulates Myosin-X. **(A)** Unfixed and unpermeabilized sections from adult mouse brains were incubated with 5 μg/ml of human monoclonal antibody (mAb) 011-138 and a commercial monoclonal mouse antibody directed against unconventional Myosin-X (Myo-X). Shown is the 3-layer cerebellar cortex consisting of the granule cell layer (GCL), Purkinje cell layer (PCL), and outermost molecular layer (ML). Incubation with mAb 011-138 resulted in staining of the Purkinje cell somata and larger blood vessels (upper panel). Staining with commercial anti-Myo-X antibody showed a comparable pattern (lower panel, no larger vessels present). Double staining revealed a high degree of signal overlap between patient 011-138 and commercial MyoX antibodies in Purkinje cells. **(B)** Likewise, double staining revealed a high degree of signal overlap between patient 011-138 and commercial Myo-X antibodies in mid-size (insets) to larger blood vessels. **(C)** Double incubation of fixed hCMEC/D3 cells with antibody 011-138 and commercial anti-Myosin-X IgG. Both stainings yielded a similar staining pattern with partially overlapping signals (see insets). **(D)** Incubation of hCMEC/D3 cells with mAb 011-38 results in the downregulation of Myo-X. Following incubation of hCMEC/D3 cells with 5 μg/ml of mAb 011-138 or mGO53 as control for 48 h cells were homogenized and subjected to Western blotting and quantitative PCR to check for protein expression and gene regulation. Incubation with mAb 011-138 decreased protein expression of Myo-X by 20% (left chart), gene regulation was unaltered (right chart). Data are given as normalized means ± SEM adjusted to loading from two individual experiments analyzed in duplicates (Western blot) and as two individual experiments (qPCR). *p ≤ 0.05.

The binding of mAb 011-138 to Myosin-X was first investigated in a cell-based assay. Expression of eGFP-tagged Myosin-X in HEK cells was verified using a commercial anti-Myo-X antibody (Figure 5C). Incubation with mAb 011-138 resulted in specific binding to transfected cells with a high degree of signal overlap between eGFP-MyoX and patient antibody signals (Figures 5D–F). Furthermore, confirmation of CSF immunoreactivity to Myo-X was received by prominent staining of transfected cells with CSF of patient 011 (Figure 5G). Further supporting our finding, patient antibody 011-138 and a commercial Myosin-X antibody stained mouse brain Purkinje cells and blood vessels in a very similar fashion with a high degree of signal overlap between both antibodies (Figures 6A,B). Incubation of hCMEC/D3 cells with commercial anti-Myo-X antibody revealed a similar, although not fully identical staining pattern (Figure 6C). Double staining with mAb 011-138 revealed a partial overlap of both signals being most prominent at the cell periphery (Figure 6C insets a and b). Expression of Myosin-X protein in hCMEC/D3 cells was verified by Western blotting (Figure 6D). Furthermore, while mRNA levels remained unchanged, we detected a decrease in Myosin-X expression at the protein level in mAb 011-138 treated cells (Figure 6D).

## Discussion

This qualitative study is the first to characterize a subgroup of brain blood vessel reactive autoantibodies in autoimmune encephalitis patients. Our data shows a range of binding patterns which point towards a bandwidth of possible target antigens. Results from our *in vivo* experiments suggest that blood vessel reactive autoantibodies are capable of binding to brain vessel epitopes when administered to the CSF. Furthermore, our results indicate –though still limited to a small number of cases- ways of potential principal contribution from blood vessel reactive antibodies to the pathomechanisms of autoimmune encephalitis as a setscrew in BBB disruption.

The growing interest in the pathomechanisms of autoimmune encephalitis has strongly fueled research efforts and thereby improved our understanding—especially of disease-defining autoantibodies targeting extracellular proteins. However, studies have shown that additional antibodies coexist in patients’ CSF. In fact, non-disease-defining autoantibodies make up the majority of the antibodies generated (Kreye et al., 2016). Furthermore, antibody-associated neurological diseases typically present with a wide range of clinical symptoms (Titulaer et al., 2013), with the result that variable clinical pictures can be associated with the same antibody. Since antibody titers only partially correlate with the clinical course (Gresa-Arribas et al., 2014), other contributing factors are suspected. Such as differential epitope specificity, strong differences in affinity, and contribution of low-affinity antibodies currently eluding diagnostic observation (Ly et al., 2018; Wagner et al., 2020). The contribution of coexisting non-disease-defining antibodies remains unresolved. Utilizing recombinant human monoclonal antibodies allowed for this study to vastly eliminate unspecific effects of serological components and attribute observed binding patterns and functional effects to single mAbs. This unbiased approach of screening mAbs on murine unfixed brain sections has proven useful in previous exploratory antibody studies (Kreye et al., 2016, 2021).

In this study, we focused on CSF-derived mAbs with reactivity against blood vessels from patients with autoimmune encephalitis. GABA_A_R and NMDAR encephalitis patient-derived mAbs exhibited diverse binding to brain blood vessels, suggesting that mAbs likely target several different antigens on the brain vasculature. Interestingly, even within the same GABA_A_ receptor encephalitis patient (113) blood vessel reactive antibodies showed differing binding patterns. This is in line with the observed variability of non-GABA_A_ receptor reactive neuronal antibodies, shown previously on murine brain tissue (Kreye et al., 2021). However, overall blood vessel reactive mAbs can be roughly divided into two “pattern groups”. One comprised of mAbs with reactivity to mid- to large size vessels, while the second group mAbs reacted to vessels of all sizes including capillaries. The binding pattern in the second group points towards a potential target that is present throughout the entire vascular tree. Since this study had an exploratory approach and investigated only a small number of patients, the frequency of blood vessel reactive antibodies in autoimmune encephalitis remains to be ascertained in future studies targeting exactly this subgroup of antibodies in larger patient cohorts.

The BBB represents a critical gatekeeper between blood circulation and brain tissue. Its function is maintained mainly by an endothelial cell layer tightly sealed by Claudins, Occludin, and junction adhesion proteins like VE-cadherin (Rubin et al., 1991; Corada et al., 1999; Vorbrodt and Dobrogowska, 2003). Human hCMEC/D3 cells are among the most commonly used and best characterized *in vitro* BBB models (Helms et al., 2016). The integrity of the endothelial cell layer was measured through transendothelial electrical resistance (TEER), which is a widely used and accepted method (Srinivasan et al., 2015; Burek et al., 2019). We found mAb 011-138 to significantly decrease TEER values in the hCMEC/D3 BBB model after treatment for 48 h in comparison to the control antibody, indicating a disruption in the BBB integrity, which was not observed when other antibodies were applied. Mab 011-138 treated cells also showed a significant decrease in Occludin expression. No significant changes were observed for other junctional proteins, amongst them VE-cadherin, which represents the main player at adherens junctions (Corada et al., 1999). Occludin is a tight junction specific protein with regulatory functions at the BBB. Among other mechanisms, altered expression of VE-cadherin and Occludin has been found to influence TEER and to associate with increased permeability of brain endothelial cells (Wang et al., 2001; Xu et al., 2012; Hebda et al., 2013; Mishra and Singh, 2013).

Our investigations *in vivo* show that intrathecally applied mAb 011-138 can reach its target in brain blood vessels. We replicated the mAb 011-138 characteristic binding pattern using patient CSF on unfixed murine brain sections. This ensures that the patient’s CSF indeed contains mAb 011-138. Our *in vivo* experiments thus replicated our *in vitro* findings and support the concept of antibody-antigen binding of mAb 011-138 to brain blood vessel targets when present in patient CSF. Moreover, in line with previous *in vitro* findings, we detected decreased Occludin expression in brain lysates of mAb 011-138 treated animals. This undermines the involvement of Occludin in the effects of antibody treatment to endothelial layer integrity. Considering this ability of mAbs to decrease electrical resistance and change the expression of junction components it could be further speculated that they might also enhance the permeability of larger molecules, although this needs to be assessed in future studies. Specifically utilizing monoclonal antibodies in the future will allow for the attribution of effects to certain mAbs.

We provide conclusive evidence to propose Myosin-X as a target of blood vessel reactive patient mAb 011-138. Myosin-X is currently the first and only known representative of unconventional myosin class X (Berg et al., 2000). It is expressed in most tissues, although at low levels, including brain cerebellar Purkinje cells (Berg et al., 2000; Sousa et al., 2006). MAb 011-138 predominantly stained mid- to large sized blood vessels in unfixed unpermeablized tissue, which initially showed binding to vascular smooth muscle cell layer rather than to endothelial cells. However, previous studies have shown Myosin-X expression and function in endothelial cells (Almagro et al., 2010). This was also reflected in our detection of Myosin-X in hCMEC/D3 cells using Western blot and qPCR analysis. Since Myosin-X is expressed at low levels, it may have evaded indirect immunofluorescence staining by patient mAb 011-138 in unpermeabilized endothelial cells. In permeabilized hCMEC/D3 cells we were able to detect a distinct staining pattern with commercially available Myosin-X antibody, resembling filamentous structures. There remains the possibility that mAb 011-138 binds to multiple types of myosin. We are aware of the fact that intracellular targets such as Myosin-X intuitively appear to be shielded from antibody binding at first glance. Nevertheless, some naturally occurring antibodies such as anti-DNA antibodies in SLE possess the ability to penetrate living cells (Noble et al., 2016). Among the diverse mechanisms by which cell-penetrating antibodies enter the cell, antibodies targeting intracellularly located synapsin in patients presenting with limbic encephalitis (Piepgras et al., 2015) were identified that utilize Fcy II/III receptor mediated endocytosis to reach their cytosolic target (Rocchi et al., 2019). Moreover, further intracellular autoimmune targets have been identified such as glutamate decarboxylase 65 (GAD65) and Amphiphysin in patients with the brainstem, extrapyramidal and spinal cord dysfunction, and in stiff-person syndrome respectively (Pittock et al., 2006; Geis et al., 2010).

Consistent with other classes of myosins, Myosin-X acts as an actin-based molecular motor. Nevertheless, it is presumed to have further functions including actin-membrane interaction due to its unique domain composition (Berg et al., 2000). Myosin-X is present at regions of highly dynamic actin such as the tips of filopodia (Berg et al., 2000; Berg and Cheney, 2002) and has been proposed as a candidate for trafficking VE-cadherin (Almagro et al., 2010). Knockdown of Myosin-X in developing kidney epithelial cells leads to delayed recruitment of junction proteins E-cadherin and ZO-1 reflected by a delayed peak transepithelial electrical resistance (Liu et al., 2012). The same study found that even after maturing, the epithelial monolayers showed a higher paracellular permeability. These findings together with our observation of reduced TEER and downregulation of Occludin and Myosin-X by antibody treatment support the concept of Myosin-X playing an important role in the dynamics and kinetics in polarized cells such as endothelial cells. As a player in cytoskeleton trafficking and membrane interactions, Myosin-X represents an exciting target structure for further investigations in the context of antibody-mediated diseases. In addition to blood vessel binding, cerebellar Purkinje cells were also targeted by mAb 011-138. This cell type exhibits a marked Myosin-X expression throughout development (Sousa et al., 2006). Functional implications, however, were not addressed in this study and are subject to further investigations.

The involvement of BBB disruption in autoimmune encephalitis has been proposed previously (Dalmau et al., 2007; Kreye et al., 2021). In other antibody-mediated neurological diseases such as NPSLE breakdown of the BBB has been verified as a key feature (Kowal et al., 2004; Huerta et al., 2006; Hirohata et al., 2014). Additionally, recent findings of anti-GRP78 antibodies in NMO and PCD-LEMS have revealed that endothelial targeting antibodies enhance the transmigration of pathogenic IgG (Shimizu et al., 2017, 2019). A proceeding endothelial activation as part of an inflammatory response as shown in these studies is a conceivable mechanism in our patient cohort, although this needs to be further investigated. Studying vessel targeting antibodies is therefore particularly interesting, as they potentially unfold new perspectives on the development, progression, and variability of autoimmune encephalitis.

In conclusion, we have identified vessel-targeting antibodies in autoimmune encephalitis patients, identified a corresponding antigenic structure and demonstrated the potential cause of the observed endothelial disruption. Therefore, our findings provide additional qualitative evidence for the concept of antibody-mediated BBB disruption as a further identified mechanism in neuroinflammatory diseases.

## Data availability statement

The original contributions presented in the study are included in the article/Supplementary material, further inquiries can be directed to the corresponding author.

## Ethics statement

The studies involving human participants were reviewed and approved by Institutional review board Charité-Universitätsmedizin Berlin (EA1/096/12). The patients/participants provided their written informed consent to participate in this study. The animal study was reviewed and approved by the Landesamt für Gesundheit und Soziales in Berlin, Germany (approval number 0078/19) and performed in compliance with relevant national and international guidelines for care and humane use of animals.

## Author contributions

LL, JK, MB, CC-G, SR, HP, and MH: contributed to the conception and design of the study. LL, JK, MB, CC-G, PB, ES-S, H-CK, DS, MS, PM, HP, and MH: contributed to the acquisition and analysis of data. LL, JK, MB, CC-G, PB, HP, and MH: contributed to drafting the text and preparing the figures. All authors contributed to the article and approved the submitted version.

## Supplementary material

Supplementary Figure 1 Co-stainings of monoclonal patient antibodies and CD31. Double stainings of mAbs 113-111, 113-126, 080-221, and 003-151 (5 μg/ml) with CD31 on unfixed murine brain sections confirm immunoreactivity of all four monoclonal antibodies to cerebral blood vessels.

Supplementary Figure 2 Tight junction proteins VE-cadherin, Claudin5, and ZO-1 are not downregulated *in vivo* by treatment with mAb 011-138. In brains from mice treated with mAb 011-138 or mGO53 (5 μg/ml) for control for 14 days, protein expression of VE-cadherin, Claudin5, and ZO-1 were unaltered. Data are given as means ± SEM adjusted to loading from four animals per condition. Immune signals from one animal per condition are exemplarily shown.
