# Supplementary figures and images for: Brain blood vessel autoantibodies in patients with NMDA and GABAA receptor encephalitis: identification of unconventional Myosin-X as target antigen
Source: Front Cell Neurosci. 2023 Jan 30;17:1077204. doi: 10.3389/fncel.2023.1077204 (PMC9922905; doi:10.3389/fncel.2023.1077204)

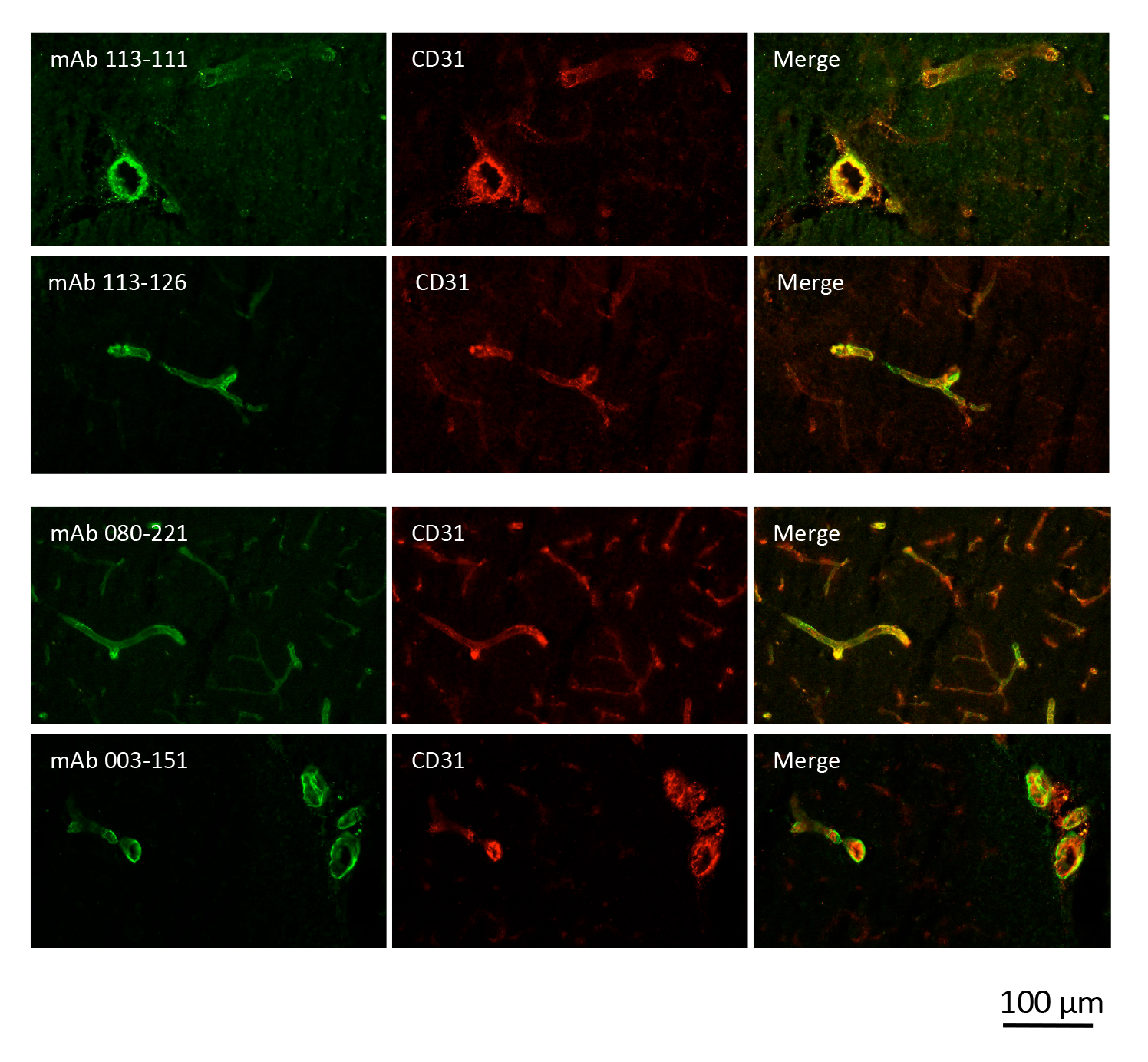

Supplement: Supplementary Figure 1 — Co-stainings of monoclonal patient antibodies and CD31. Double stainings of mAbs 113-111, 113-126, 080-221, and 003-151 (5 μg/ml) with CD31 on unfixed murine brain sections confirm immunoreactivity of all four monoclonal antibodies to cerebral blood vessels. [file Image_1.TIF]

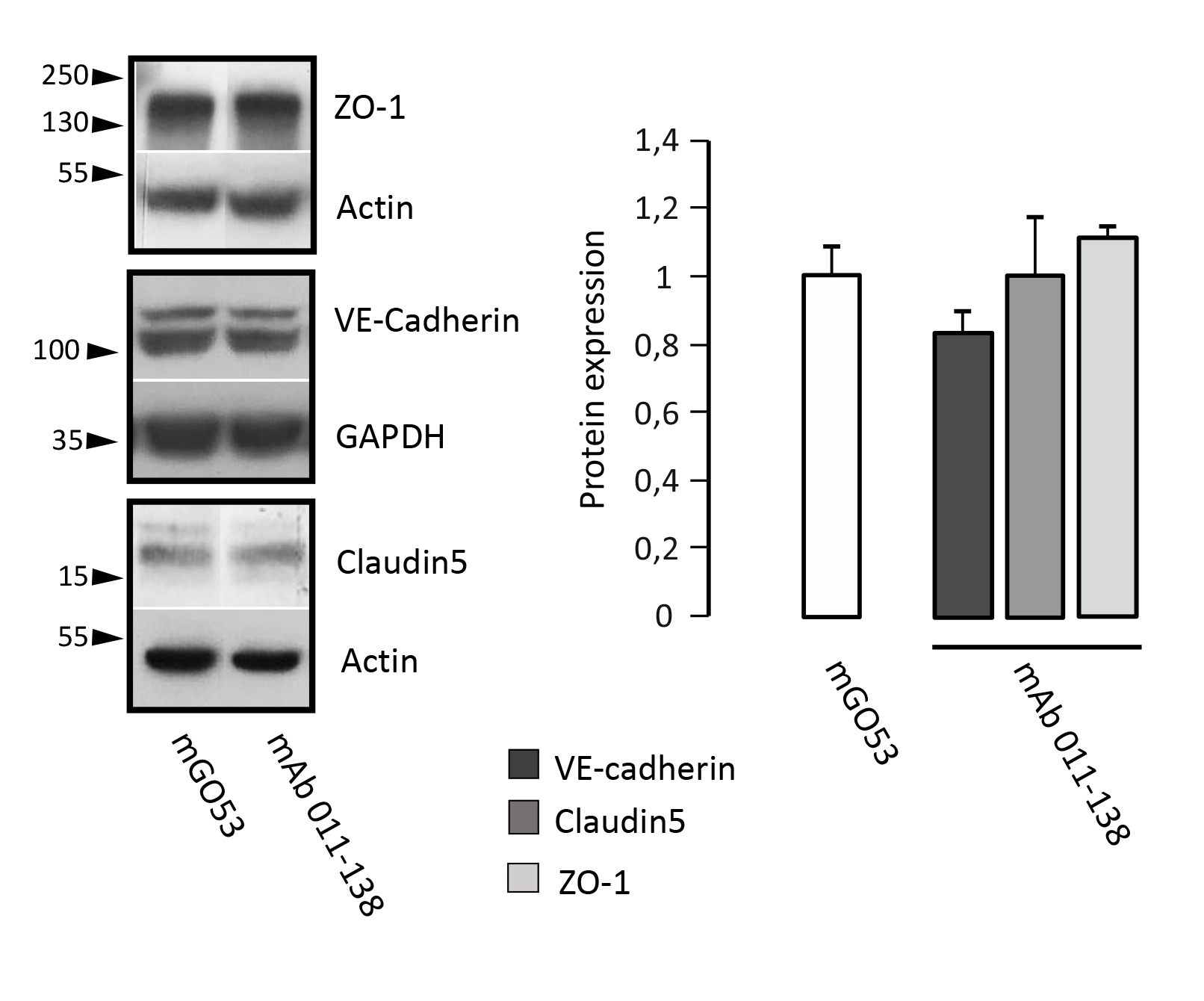

Supplement: Supplementary Figure 2 — Tight junction proteins VE-cadherin, Claudin5, and ZO-1 are not downregulated in vivo by treatment with mAb 011-138. In brains from mice treated with mAb 011-138 or mGO53 (5 μg/ml) for control for 14 days, protein expression of VE-cadherin, Claudin5, and ZO-1 were unaltered. Data are given as means ± SEM adjusted to loading from four animals per condition. Immune signals from one animal per condition are exemplarily shown. [file Image_2.TIF]
